# Neutrophil extracellular traps and the dysfunctional innate immune response of cystic fibrosis lung disease: a review

**DOI:** 10.1186/s12950-017-0176-1

**Published:** 2017-12-28

**Authors:** Sheonagh M. Law, Robert D. Gray

**Affiliations:** MRC Centre for Inflammation Research, The Queen’s Medical Research Institute, 47 Little France Crescent, Edinburgh, EH16 4TJ UK

**Keywords:** Cystic fibrosis, Neutrophil extracellular traps, NETs, Neutrophils, Macrophages, Inflammation, DNase

## Abstract

**Background:**

Cystic Fibrosis (CF) is a devastating genetic disease characterised primarily by unrelenting lung inflammation and infection resulting in premature death and significant morbidity. Neutrophil Extracellular Traps (NETs) are possibly key to inflammation in the disease. This review aims to draw together existing research investigating NETs in the context of a dysfunctional innate immune system in CF.

**Main body:**

NETs have a limited anti-microbial role in CF and studies have shown they are present in higher numbers in CF airways and their protein constituents correlate with lung function decline. Innate immune system cells express *CFTR* and myeloid-specific *CFTR* KO mice have greater neutrophil recruitment and higher pro-inflammatory cytokine production to both sterile and bacterial inflammatory challenges. *CFTR* KO neutrophils have impaired anti-microbial capacity and intrinsic abnormalities in the pH of their cytoplasm, abnormal protein trafficking, increased neutrophil elastase and myeloperoxidase function, and decreased hypochlorite concentrations in their phagolysosomes. Furthermore, neutrophils from CF patients have less intrinsic apoptosis and may be therefore more likely to make NETs. *CFTR* KO macrophages have high intraphagolysosomal pH and increased toll-like receptor 4 on their cell surface membranes, which inhibit their anti-microbial capacity and render them hyper-responsive to inflammatory stimuli, respectively. Pharmacological treatments for CF target these intrinsic abnormalities of immune dysfunction. Emerging evidence suggests that the absence of *CFTR* from neutrophils affects NETosis and the interaction of NETs with macrophages.

**Conclusion:**

Current evidence suggests that NETs contribute to inflammation and lung destruction rather than working effectively in their anti-microbial capacity. Further studies focussing on the pro-inflammatory nature of NET constituents are required to identify the exact mechanistic role of NETs in CF and potential therapeutic interventions.

## Background

Cystic Fibrosis (CF) is an autosomal recessive disorder caused by mutations in the CF transmembrane conductance regulator (*CFTR)* gene, which encodes a transmembrane anion channel transporting chloride and bicarbonate. CF may give rise to the clinical manifestations of chronic airway inflammation and infection, pancreatic insufficiency causing malnutrition and diabetes mellitus, gastrointestinal disease, and male infertility.

Presently around 11,000 people in the UK suffer from CF and the median age of death is only 28 [1], with one third of patients dying whilst waiting for a lung transplant as morbidity and mortality principally relate to chronic airway infection and inflammation [2]. A dysfunctional innate immune response is key to the lung destruction seen. In some ways, CF can be compared to an autoimmune disease in which the exogenous antigen is identified as pathogens in the airways. It has been known for over 40 years that CF patients have high levels of DNA in their airways, of which Neutrophil Extracellular Traps (NETs) have recently emerged as the principal source [3, 4].

Here we aim to review the role of NETs in CF lung disease in the context of a dysfunctional innate immune system and discuss the importance of better understanding the cellular interactions, in order to find new anti-inflammatory therapies for CF patients.

## Main body

### The anti-microbial capacity of neutrophils is impaired in the context of CFTR dysfunction

CF lung disease is characterised by a neutrophil-dominant inflammation, with neutrophils accounting for 80% of the total cells present in CF sputum, even when patients are clinically stable [5]. Although uncommon, CF provides a strong model with which to investigate the pro-inflammatory potential of NETs, which may also be informative of other neutrophil-dominated diseases such as Chronic Obstructive Lung disease, non-CF Bronchiectasis and Pneumonia. Neutrophils employ a number of antimicrobial strategies such as phagocytosis, the generation of Reactive Oxygen Species (ROS), degranulation, and NET formation, all of which have been described as abnormal in CF [6–9].

*CFTR* ^−/−^ neutrophils have impaired transport of chloride into the phagolysosome from the cytoplasm and accordingly have increased cytosolic concentrations of Na^+^ and Cl^−^ and reduced Cl- concentrations in their phagolysosome [7]. This leads to impaired destruction of pathogens because chloride, alongside hydrogen peroxide, is the substrate for hypochlorite, a potent ROS found within the phagolysosome and responsible for microbial digestion [10]. The abnormal pH of *CFTR* ^−/−^ neutrophils’ cytoplasm leads to excessive degranulation of azurophilic granules, thus increasing the release of anti-microbial enzymes such as peroxidases (e.g. myeloperoxidase, MPO), proteases (e.g. neutrophil elastase, NE) and anti-microbial peptides [11]. If there are high extracellular levels of NE, it overwhelms the binding capacity of the anti-protease α1-antitrypsin [12], and degradation of elastin within lung parenchyma contributes to lung destruction and bronchiectasis seen in CF [13]. The abnormal cytosolic ion concentrations also cause inactivation of Rab27a – a protein crucial to granule trafficking – and in turn, defective degranulation and bacterial killing [8]. Pohl et al. demonstrated that this defect was corrected by treatment of G551D CF patients with Ivacaftor (an ion channel potentiator) [8].

We have recently shown that *CFTR* ^−/−^ neutrophils have a primary defect causing decreased spontaneous apoptosis and increased levels of NET formation that can promote inflammation through their interactions with macrophages [9]. This defect in apoptosis leading to increased neutrophil survival in CF has been described by other authors [14, 15] and may be detrimental to the host due to defective resolution of infectious or inflammatory insults.

### Neutrophil extracellular traps

**Fig. 1:**
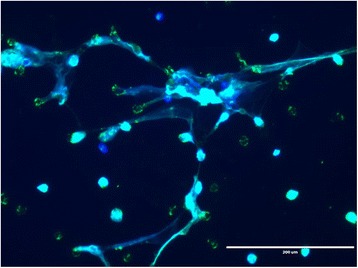
Overlay of immunofluorescence images demonstrating co-localisation of DNA and myeloperoxidase (MPO) on in vitro NETs stained with Hoechst antibody (blue, binds DNA) and anti-MPO antibody (green)

NET formation, or “NETosis”, may be the favoured cell death process by *CFTR* ^−/−^ neutrophils, over apoptosis. NETosis can be triggered by neutrophils undergoing oxidative burst [16] leading to enzyme-mediated decondensation of nuclear chromatin, rupture of the nuclear membrane and release of chromatin and cellular proteins into the extracellular space. NETs form web-like structures composed of de-condensed Deoxyribonucleic acid (DNA), citrullinated histones and several pro-inflammatory cytosolic and granule proteins including calprotectin, NE, MPO and LL37 [17, 18]. A spatial relationship between DNA and these proteins is a key requirement to differentiate NETs from fibrin or bacterial DNA in vitro [18]. Figure 1 below shows an immunofluorescence image of NETs in vitro*.*

### NETs may be a key source of inflammation in CF lung disease

NETs were initially described in 2004 as an anti-bacterial defence mechanism [19] but their potential as a driver of inflammation in CF has yet to be investigated and may provide a therapeutic target in lung disease. In particular, the interaction of NETs with other immune cells and lung epithelial cells may be of key importance. Throughout this review, we will consider NETs in the context of the dysfunctional innate immune system in CF, which is characterised by frustrated inflammation.

Original studies demonstrated that NETosis was a form of cell death distinct from apoptosis, potentially utilised as a last resort in neutrophils’ anti-microbial defence strategies. However other evidence has suggested that neutrophils may also release NETS in vivo without cell death in a process termed “vital” NETosis [20]. Furthermore, vital NETosis can occur by release of mitochondrial DNA [21], which does not require the presence of citrullinated histones on the DNA backbone [9]. Whether NETosis with cell death and vital NETosis represent the same or different biological phenomena remains unclear at present [19, 22].

**Fig. 2:**
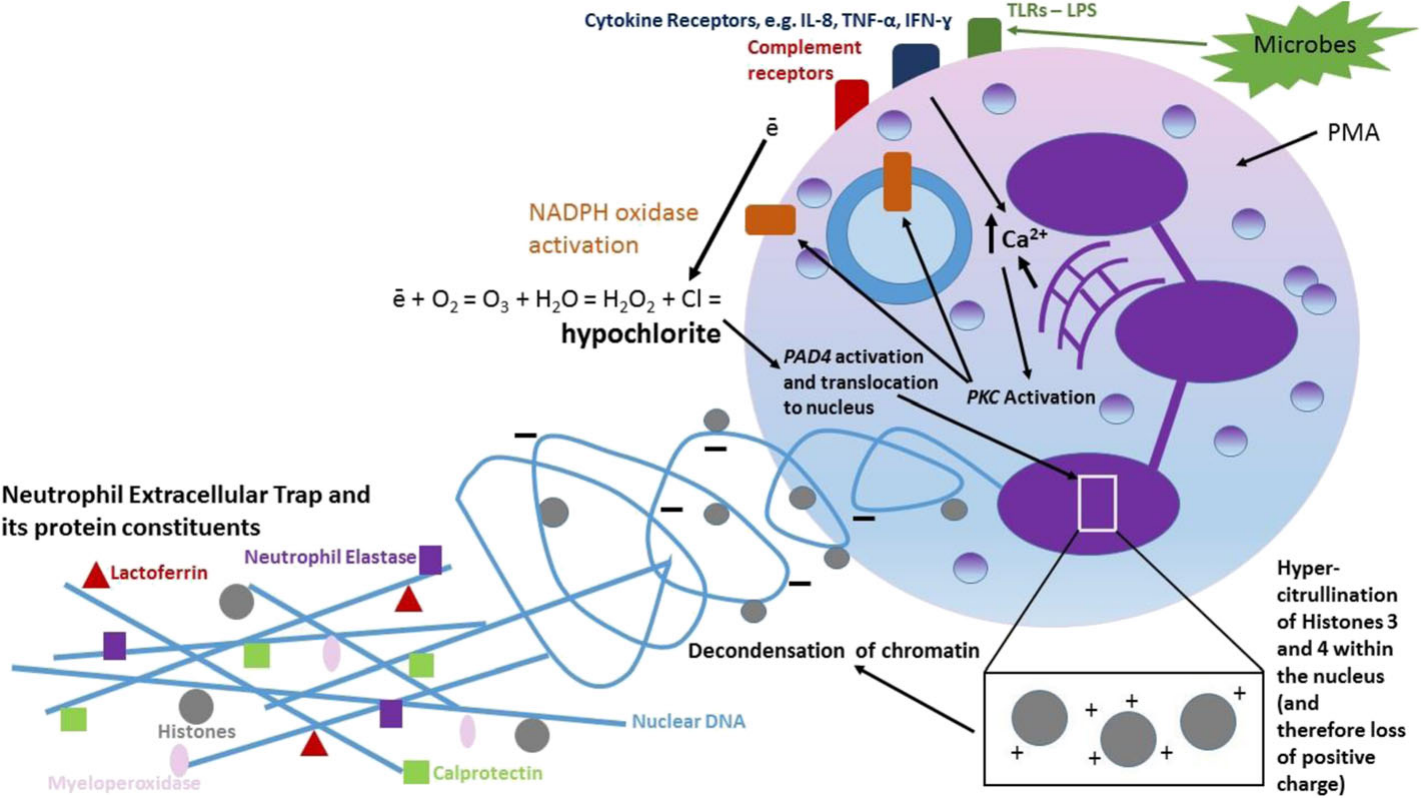
Neutrophil Extracellular Trap (NET) production by a neutrophil. Neutrophils are stimulated to form NETs by several microbial, inflammatory and sterile endogenous triggers. These bind onto cell surface receptors including Toll-like Receptor 4, cytokine and complement receptors. Receptor binding leads to increased calcium release from the endoplasmic reticululm, activating Protein Kinase C (PKC). This leads to activation of NADPH Oxidase on the cell membrane and lysosomes, forming superoxide which reacts with water and chloride to form hypochlorite. Hypochlorite activates Protein Arginine Deiminase 4 (PAD4) which translocates to the nucleus where it catalyses hypercitrullination of histones 3 and 4 [26]. This causes the histones to lose their positive charge and in doing so weakens their binding to DNA, leading to decondensation of chromatin. There is loss of plasma membrane integrity then decondensed chromatin and histones are expelled into the extracellular space where they form complexes with granule/cytosolic proteins such as myeloperoxidase, neutrophil elastase and calprotectin. Recent research suggests NET production is an end-point of numerous cell signalling pathways – not all of which require each of the above steps – dependent upon the stimulant used to induce NETosis [25].

**Fig. 3:**
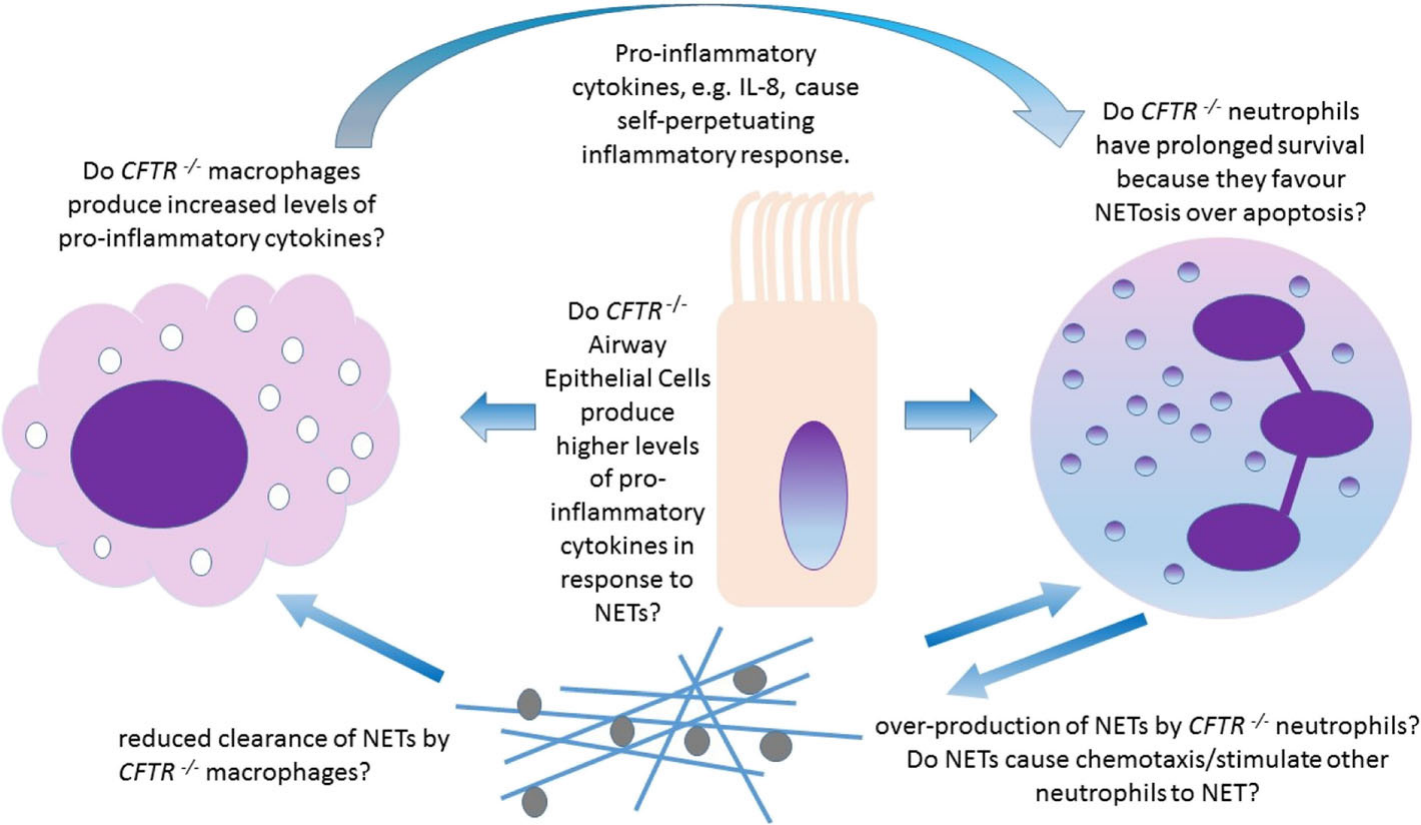
Several mechanisms require investigation concerning the dysfunctional innate immune response and NETs in Cystic Fibrosis (CF). It may be that CFTR ^−/−^ neutrophils are hyper-stimulatory to macrophages due to increased NET production and CFTR ^−/−^ alveolar macrophages produce higher levels of pro-inflammatory cytokines, driving chemotaxis of more neutrophils in a self-perpetuating cycle of inflammation. Co-culture experiments with airway epithelial cells, NETs and macrophages would mimic in vivo conditions. Targeting this cycle, e.g. by reducing NET production, inhibiting NET-protein’s deleterious functions or promoting NET clearance would provide new targets for the treatment of CF

NETosis is triggered by microbial (bacterial, fungal and viral), inflammatory (e.g. IL-8, IFN-ɣ, TNF-α), and endogenous “sterile” triggers (e.g. nitric oxide, platelets, complement, monosodium urate crystals) [23]. An elegant summary of the molecular mechanisms regulating NETosis is described in the recent review by Papayannopoulos [24]. Figure 2 shows the intracellular pathways understood to be involved in NETosis, although Kenny et al. recently demonstrated that NETosis can occur through numerous signalling mechanisms, dependent upon the stimulant to which neutrophils were exposed [25] (Fig. 3).

### NETs have a limited anti-microbial role in CF

NETs have been conserved throughout evolution for their anti-microbial role. Indeed extracellular trap formation has been demonstrated as critical for encapsulation of microbes by haemocytes of the shore crab *Carcinus maenas* [27], and NETs (or equivalent) have been described in fish [28], chickens [29], dogs [30] and pigs [31]. Studies in humans have also demonstrated NETs to be anti-microbial [20]. Whilst laboratory strains and CF clinical isolates of *Pseudomonas Aeruginosa (P.Aeruginosa)* strongly trigger NET release [32], late isolates within CF airways acquire resistance against NETs [33, 34]. Furthermore, Fuchs et al. showed that only 25% of neutrophils form NETs to kill *S. Aureus* in vitro*;* the majority of bacterial killing was by phagocytosis [16]. NETosis appears better-suited to tackling large microbes, such as fungal hyphae, which are too large to be phagocytosed [35]. Marcos et al. found NETs in airway samples of CF patients were associated with fungal colonisation with *Aspergillus Fumigatus (A. Fumigatus)* but not with bacterial infection [4].

We hypothesise that in the context of CF, NETs could play only a minor anti-microbial role, as despite the presence of large numbers of activated neutrophils and extracellular DNA, patients suffer recurrent lower respiratory tract infections and colonisation with organisms such as Staphylococcus Aureus (*S. Aureus), P. Aeruginosa* and *A. Fumigatus* as the host’s defence mechanisms are overwhelmed.

NETs can be thought of as the Jekyll and Hyde of immunity; on one hand, they fulfil their bacteriostatic and bactericidal roles in lower order species beautifully [25] but on the other hand, due to the presence of an adaptive innate immune system, function as detrimental auto-antigens in human autoimmune disease.

### NETs contribute to autoimmune disease and sterile inflammation

There are a number of diseases characterised by NET-mediated sterile inflammation including Systemic Lupus Erythematosus (SLE), psoriasis, small vessel vasculitis, rheumatoid arthritis, gout, venous thrombosis and cardiovascular disease [23, 36–39]. The autoantibodies used by clinicians to diagnose some of these conditions e.g. anti-MPO, anti-Proteinase 3, extractable nuclear antibodies, anti-nuclear antibodies and anti-dsDNA antibodies – all target NET-bound proteins. SLE patients unable to clear NETs suffer renal impairment secondary to lupus nephritis [36]. Additionally, during the pathophysiology of atherosclerosis, NETs activate macrophages to release pro-inflammatory cytokines which in turn activate T helper 17 cells, driving further immune cell recruitment and exacerbating inflammation [39].

It follows that NETs may be an important pro-inflammatory component in the CF airway, where inflammation is driven by chronic infection.

### The pathophysiological role of NETs in CF

Higher levels of free DNA are present in bronchoalveolar lavage (BAL) samples of CF patients compared to healthy controls [40]. Originally it was thought this DNA was derived from apoptotic and necrotic cell debris but recent studies have suggested most arises from NETs [3, 4]. NETosis can be triggered by *P. Aeruginosa* [41]*,* a major pathogen in CF lung disease, and several studies have demonstrated that NETs are found in higher levels in airway samples from CF patients compared to healthy controls and are associated with poorer lung function [3, 4]. DNase is a treatment used mainly in adult CF patients and we hypothesise it targets NETs. It is a nebulised therapy used to improve patients’ lung function and reduce exacerbation rate in both paediatric and adult patients [42–44]. It functions to cleave the excessive extracellular DNA to decrease sputum viscosity [45] but also reduces airway inflammation [46].

This DNA backbone of NETs is the scaffolding upon which several pro-inflammatory proteins reside. These have been implicated in the pathophysiology of lung injury and inflammation and key proteins shall now be discussed in turn.

### Histones

Histones, for example, are known to cause lung injury in mice [47–50] in part through charge-mediated binding and disruption of phosphodiester bonds within cell membranes, thus causing toxic calcium influx [47] and through TLR2 and TLR4 binding [49]. Histone sub-types - H1, H2A, H2B, H3 - have defined effects upon murine and human phagocytes; they can lead to cell swelling, lactate dehydrogenase release, cytokine and chemokine release to varying degrees [49]. The treatment of mice with DNase has been shown to reduce citrullinated H3 levels in BAL fluid and protect from Lipopolysaccharide (LPS)-induced acute lung injury [50].

### Neutrophil Elastase (NE)

NE is another key NET constituent implicated in the pathophysiology of CF. It is an azurophilic granule protein functioning to degrade phagocytosed proteins. Within the airways, NE degrades protein structures including elastin and collagen [6]. It is found in high concentrations in CF sputum and BAL samples and levels correlate with lung function decline in CF [13, 51]. Dubois and co-workers demonstrated that in vitro DNase treatment significantly increased NE activity [52]. They concluded that negatively charged DNA binds to anionic NE and in doing so protects it from proteolytic degradation by anti-proteases in the lungs. This interaction likely contributes to the protease-anti-protease imbalance seen in CF lung disease, which leads to parenchymal destruction.

### Calprotectin

Calprotectin, a neutrophil cytosolic protein, is also incorporated onto NETs during NETosis and has received renewed interest in the field of CF. We have demonstrated that Calprotectin could be used as a biomarker for CF. Both sputum and serum calprotectin levels significantly decrease following treatment of an exacerbation, and serum calprotectin levels were negatively correlated with Forced Expiratory Volume in one second (a measure of patient lung function) and predicted time to next exacerbation [53]. Calprotectin also promotes inflammation by functioning as an alarmin [54]. Calprotectin on NETs has been shown to be crucial as an anti-fungal defence in mice [55] but the potential for NET-bound calprotectin to promote inflammation is yet to be investigated.

### Bactericidal permeability-increasing protein (BPI)

BPI is an antimicrobial peptide, which targets gram-negative bacteria and is stored in azurophilic granules of neutrophils. It becomes localised onto NETs following PMA-induced NET formation [56]. CF patients develop anti-BPI autoantibodies, levels of which negatively correlate with lung function [56]. This gives further evidence that NETs are involved in autoimmunity in CF.

Some of these pro-inflammatory proteins have been visualised using the recently developed intra-vital microscopy, whereby biological cellular processes are imaged in living animals, a technique revolutionising the study of immunology [57]. Kolaczkowska et al. used intra-vital microscopy to image Methicillin-resistant S. Aureus (MRSA)-induced NETs and their associated NE and MPO within the liver sinusoids of mice [58]. They demonstrated that blocking NET formation protected the liver from MRSA-induced damage, suggesting NETs, not bacteria, were responsible for the damage observed [53].

Given that the above proteins found upon NETs correlate with lung function decline in CF and cause inflammation, it is possible that NETS are involved in the pathophysiology of CF, although mechanisms require further elucidation. We hypothesise lack of *CFTR* causes abnormal function of innate immune system cells leading to excessive production and/or impaired clearance of NETs. Additionally, we have recently shown that CF neutrophils live longer due to decreased apoptosis and produce more NETs compared to healthy controls [9], although the underlying mechanisms require further investigation.

### Neutrophils express CFTR and are dysfunctional in CF

A simple explanation for the abnormal accumulation of NETs in CF in might be that there are higher numbers of neutrophils within the airways. Alternatively, *CFTR* ^*−/−*^ neutrophils may produce excessive amounts of NETs via abnormal intracellular pathways. Painter et al. used reverse transcriptase-polymerase chain reaction, immunofluorescence staining, and immunoblotting to demonstrate low level expression of CFTR in neutrophils at both mRNA and protein levels, with CFTR present in secretory vesicles and phagolysosomes [7], suggesting it could have a number of roles in neutrophil function. Bonfield et al. bred whole-body and myeloid-specific *CFTR* inactivated mice and challenged them with *P. Aeruginosa* lung infection: *CFTR* ^*−/−*^ mice had increased neutrophil numbers, IL-1β and IL-6 in BAL at day 10 post-infection [59]. The same was seen for myeloid cell *CFTR* ^−/−^ mice, albeit to a lesser extent [59]. Su et al. demonstrated that a lack of functional CFTR in neutrophils (either by pharmacological inhibition or knock-out chimera model) promoted LPS-induced lung inflammation and injury in mice [60]. Taken together, these three studies support the view that dysfunctional neutrophil CFTR contributes to lung inflammation in CF, although do not implicate NETs directly.

Evidence is sparse with regard to intrinsic abnormalities in NETosis in CFTR ^−/−^ neutrophils. One study specifically addressing defects in NETosis was that by Akong-Moore et al.*,* who performed in vitro experiments stimulating neutrophils to form NETs using PMA in the presence and absence of chloride in the culture media. They found NETosis was significantly decreased in the absence of extracellular chloride [61], which may be key in CF.

### Intrinsic abnormalities of CF macrophages may contribute to accumulation of NETs in CF airways

Clearance of NETs from the airways is dependent upon several factors including mucociliary clearance, DNase activity and phagocytosis by macrophages, which phagocytose NETs then digest them within lysosomes, a process facilitated by pre-degradation by DNase and one which does not result in pro-inflammatory cytokine secretion [62]. This process may be deficient in CF given that CFTR-deficient or *CFTR* ^−/−^ macrophages have abnormally high intraphagolysosomal pH, which was shown to impair bactericidal activity [63].

Furthermore, Bruscia et al. have shown that *CFTR* ^−/−^ macrophages have significantly higher levels of Toll-like Receptor 4 (TLR 4) on plasma cell membranes compared to wild-type macrophages [64]. TLR 4 is a protein that recognises pathogen-associated molecular patterns such as that from LPS, found on the cell membranes of Gram-negative bacteria. They found that in response to LPS challenge, CF macrophages produced significantly higher amounts of the pro-inflammatory cytokines TNF-α, IL-6 and GMCSF, demonstrating CF macrophages have a hyper-responsive phenotype [64]. This may be via the NFκB and mitogen-activated protein kinases (MAPK) cell signalling pathways, which increase gene transcription of pro-inflammatory cytokines, including IL-8 [64, 65], a potent chemoattractant for neutrophils. Interestingly, CFTR has recently been shown to prevent macrophage-driven inflammation and atherogenesis in apolipoprotein E-deficient mice, via inhibition of NFκB and MAPK activation [66]. To conclude, intrinsic abnormalities of CF macrophages contribute to the chronic neutrophilic inflammation seen in CF lung disease due to their hyper-responsive phenotype to inflammatory stimuli.

### Pharmacological treatments for CF target these intrinsic abnormalities of immune dysfunction

Aforementioned macrophage and neutrophil dysfunctions are the target of treatments given to CF patients. For example, the macrolide antibiotic Azithromycin functions in an immunomodulatory capacity to reduce pro-inflammatory cytokine production from alveolar macrophages and is known to reduce pulmonary exacerbation rates and promote weight gain in CF [67, 68]. Ivacaftor, a CFTR potentiator, corrects neutrophil Rab27a trafficking and normalises cytosolic Na^+^ and Cl^−^ concentration abnormalities in vitro [8]*.* The decreased apoptosis we demonstrated in CF neutrophils was reversed by Ivacaftor in patients with gating (G551D) mutations [9].

Cyclin-dependent kinase (CDK) proteins are involved in cell cycle control and are now believed to play a role in immunity; CDK 4 and 6 have recently been shown to regulate NET formation [69]. (R)-Roscovitine, a CDK inhibitor, is a drug currently under investigation in the field of CF. It has been shown to: 1) restore normal pH within phagolysosomes of CF alveolar macrophages; 2) induce neutrophil apoptosis, which is impaired in CF neutrophils; and 3) act as a CFTR modulator by reducing DF508-CFTR degradation and aiding its transfer to the plasma membrane [70]. Interestingly, the phase II study entitled “Evaluation of (R)-Roscovitine Safety and Effects in Subjects With Cystic Fibrosis, Homozygous for the F508del-CFTR Mutation (ROSCO-CF)” is ongoing to assess the safety of increasing doses of (R)-Roscovitine in adult CF subjects chronically infected with Pseudomonas aeruginosa [71]. Whilst not specifically targeting NETs, this drug represent a future pharmacological treatment for CF patients.

This review has presented a strong argument for dysfunctional host innate immune responses contributing to lung injury in CF. What remains uncertain is the interplay between NETs and macrophages in CF – NETs may be hyper-stimulatory and macrophages hyper-responsive.

### Suggestions for future work

This literature review has identified gaps in our knowledge regarding the interplay between NETs and innate immune system cells, which contributes to the pathophysiology of CF. Whether the higher levels of NETs in CF airways samples is the result of over-production by the *CFTR* ^−/−^ neutrophil, increased survival of *CFTR* ^−/−^ neutrophils or reduced clearance by alveolar macrophages, requires investigation. We do not know the cell signalling pathways causing *CFTR* ^−/−^ neutrophils to have prolonged survival. There is a paucity of data on how absence of CFTR affects NETosis pathways. From a clinical perspective, it may be that DNase should be introduced as a therapeutic agent earlier in disease than present practice, thus reducing inflammation caused by the dysfunctional immune system, even before chronic infection is established.

## Conclusions

CF is a devastating genetic disease resulting in premature death of patients, usually because of respiratory failure. The dysfunctional innate immune response, specifically the interplay between neutrophils and macrophages, may have an important role in the pathophysiology of CF lung disease. We hypothesise NETs are a hindrance rather than a help in CF, contributing to inflammation and lung damage rather than working effectively in their anti-microbial capacity. Further studies are essential to investigate the pro-inflammatory nature of NET constituents with the aim of identifying precise, new treatment strategies for CF.

## Abbreviations

*A. Fumigatus*: Aspergillus Fumigatus
BAL: Bronchoalveolar lavage
CDK: Cyclin Dependent Kinase
CF: Cystic Fibrosis
CFTR: Cystic Fibrosis Transmembrane Conductance Regulator
DNA: Deoxyribonucleic Acid
LPS: Lipopolysaccharide
MAPK: Mitogen-activated protein kinase
MPO: Myeloperoxidase
MRSA: Methicillin-resistant *Staphylococcus Aureus*
NE: Neutrophil Elastase
NETs: Neutrophil Extracellular Traps
*P. Aeruginosa*: Pseudomonas Aeruginosa
PKC: Protein Kinase C
PMA: Phorbol Myristate Acetate
ROS: Reactive Oxygen Species
*S. Aureus*: Staphylococcus Aureus
TLR 4: Toll-like receptor 4
